# Necking of epithelial tissues with cellular topological transition

**DOI:** 10.1371/journal.pcbi.1014565

**Published:** 2026-07-29

**Authors:** Yuan He, Shi-Lei Xue

**Affiliations:** 1 School of Materials Science and Engineering, Zhejiang University, Hangzhou, Zhejiang, China; 2 Department of Materials Science and Engineering, School of Engineering, Westlake University, Hangzhou, Zhejiang, China; Indian Institute of Technology Hyderabad, INDIA

## Abstract

As the cover of embryos and adult organisms, epithelial tissues are subjected to substantial mechanical forces in tissue morphogenesis. However, the finite deformation behaviors of epithelial tissues remain largely unexplored. This study combines discrete vertex simulations with an analytical constitutive model to investigate the necking behavior of epithelial tissues. In this model, the shape changes and topological transitions of single cells are associated with the elastic and inelastic components of tissue deformation via a mean-field formulation. Our results show that the necking bifurcation of a stretched tissue strongly associated with cellular topological transitions. The bifurcation condition and the steady state of necking propagation are predicted from the constitutive model and validated by vertex simulations. Furthermore, we find that topological defects in disordered tissues facilitate necking bifurcation but impede its propagation. These defects also induce the necked region to collapse into a thin thread, as observed in real tissues. Finally, our simulations show that necking also occurs under various tissue surface tensions and configurations (e.g., tubular geometry), demonstrating the generality of this behavior. Together, our work provides valuable insights into the deformation behaviors of epithelial tissues.

## Author summary

Epithelial tissues form thin sheets that protect organs and play key roles in development and disease. During morphogenesis and mechanical loading, these tissues often undergo large deformations, including narrowing, thinning, and eventual rupture. Although experiments have observed striking behaviors such as tissue necking, the cellular mechanisms that drive these large-scale mechanical instabilities remain unclear. Here, we combine a computational vertex model with an analytical theoretical framework to investigate necking in epithelial tissues under stretching. Our approach explicitly links tissue-scale deformation to cell-scale mechanical events. We show that reversible changes in cell shape produce elastic tissue deformation, whereas irreversible cell rearrangements, known as topological transitions, are associated with inelastic deformation. Importantly, we find that tissue necking is enabled by these cellular rearrangements rather than by elastic deformation alone. Our model predicts when necking initiates, how it propagates through the tissue, and how stress relaxes during deformation, and these predictions are quantitatively validated by cell-based simulations. We further demonstrate that topological defects in disordered tissues strongly influence where necking begins and whether it propagates. Moreover, necking behavior is robust under various tissue surface tensions and configurations. Together, our results provide a mechanistic link between cell rearrangements and large-scale mechanical instabilities in epithelial tissues, offering insights into tissue morphogenesis and mechanical failure.

## Introduction

Epithelial tissues are one-cell-thick continuous sheets that cover the surfaces of embryos and adult organisms. Recently, mechanics of epithelial tissues have been found to be important for tissue morphogenesis. Subjected to various mechanical forces, epithelial tissues can undergo remarkably large-scale deformations and endow organisms with diverse morphologies [1,2]. For instance, active contractile forces arising from cellular actin-myosin network can drive the in-plane or out-of-plane deformations of epithelial tissues, as observed in *Drosophila* mesoderm invagination [3], intestinal crypt formation [4], vertebrate neurulation [5,6], and so on. Mechanical compression from neighboring tissue compartments is responsible for the undulated tissue surfaces of mucosa [7,8], intestine [9–11], and brain cortex [12–14]. Hydrostatic pressure, arising from cellular osmotic regulation, has also been proven to power lumen formation and tissue expansion in a variety of settings [15–17].

**Fig 1:**
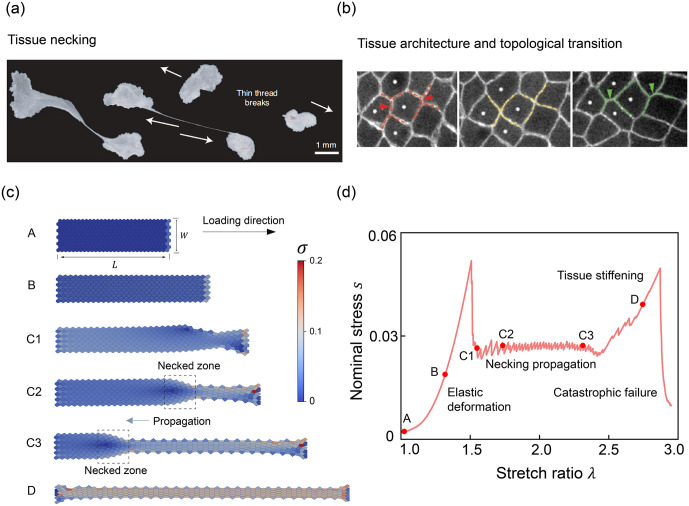
Tissue necking, cellular topological transition and discrete vertex simulation of uniaxial tissue stretching. **(a)** Necking instability during the body elongation of *Trichoplax adhaerens*, a simple marine animal mainly composed of epithelial tissues (Prakash et al., 2021). From left to right, the necked region gradually propagates until final breakage. **(b)** Representative topological transition event at the cell scale in the epithelial tissue from a *Drosophila* embryo (Bertet et al., 2004): in the four-cell unit, the cell-cell junctions (red arrows) first shrink into points, then elongate perpendicular to the original direction (green arrows). After this transition, cells change their positions and neighbors. **(c)** Representative snapshots of the tissue at different stages: (A) initial stress-free state, (B) elastic deformation, (C1-C3) necking bifurcation and propagation, and (D) tissue stiffening. The spatial distributions of the uniaxial Virial stress σ are shown in each snapshot. **(d)** The resulting nonlinear stress-stretch response of the epithelial tissue under uniaxial tension.

Epithelial tissues have been found to show non-trivial deformation features. An interesting example is the body elongation of a marine animal *Trichoplax adhaerens*, whose self-motility drives remarkable morphological changes of its own epithelial tissue: the originally disk-like epithelial sheet forms holes and further breaks into thin, long threads [18]. Notably, with persistent stretching, the tissue thread becomes thinner in the middle and forms a necked region there, which propagates along the thread until the final breakage (Fig 1a). Related necking-like and failure behavior have also been observed *in vitro.* Wang [19] observed necking and failure of cardiac microtissues cultured in fibrin or collagen gels, where the contractility of cardiac cells causes localized thinning, leading to tissue failure via narrowing and subsequent elongation. Lv [20] reported active hole formation in epithelial monolayers, where multicellular bridges between neighboring holes underwent localized necking and eventual rupture. Such tissue deformation behavior is reminiscent of the necking instability in metals [21], metallic glasses [22], glassy polymers [23] and soft elastomers [24]. Considère [25] first identified that the necking bifurcation of ductile materials occurred when the external load reached its maximum. Since then, the necking bifurcation of various materials has been studied extensively [26–30]. However, the necking phenomenon in soft tissues remains largely unexplored. An epithelial monolayer is made of polygon-like cells that tightly bind via cell-cell junctions [5,31]. Importantly, the cell edges can remodel (i.e., new edges can be created while existing ones can be annihilated) and the final cell shape is a result of the competition among the adhesion forces at the cell-cell junctions, fluid pressure of its cytoplasm, and contractile forces of its actomyosin networks near the cell-cell interface [5,32]. Such edge remodeling drives cell shape changes and rearrangements (Fig 1b), which collectively determine how the tissue deforms at the macroscopic scale. Thus, to uncover the physical principle underlying the non-trivial tissue deformation like tissue necking, the individual cellular events should be taken into account.

Cell-based discrete tissue models, which capture basic mechanical and geometric properties of individual cells in soft tissues, have been developed to better understand how the mechanics and morphology of single cells affect tissue-scale deformation and morphogenesis. Representative cell-based models include the vertex model [33–37], cellular Potts model [38,39], and particle-based models [40–42]. Among them, the vertex model is particularly attractive for the simulation of epithelial tissues, due to its ability to well capture the confluency of epithelial tissues, as well as key mechanical cues (such as cell adhesion and contractility) of individual cells and the remodeling of the cell-cell interface. The vertex modeling has successfully uncovered interesting features of epithelial tissues, such as cell packing irregularity [43] and fluid-solid rigidity transition [44]. However, most of the previous studies are limited to infinitesimal tissue deformation [45–48]. For instance, Bi [46] found that enhanced cell-cell adhesion (or weakened cell contractility) can drive a transition in which the tissue’s instantaneous modulus softens to zero. On the other hand, there have been efforts to formulate the continuum models of epithelial tissues by combining ingredients from the vertex model and continuum field theories [45,49–52]. These approaches have successfully captured tissue-scale mechanical evolutions and features such as rigidity transitions [45,52], strain stiffening at intermediate strains [50] and the effects of cell rearrangements in tissue plastic flow [49,50] through internal variables. However, they ignore the details and the stochastic nature of individual cell events, thus failing to fully depict the versatile mechanical behaviors of epithelial tissues or provide direct analytical predictions for discrete vertex-model simulations.

Here, we combine an analytical constitutive model (i.e., a closed-form stress-strain relationship) with discrete vertex simulations to investigate the necking instability emerging in epithelial sheets. The constitutive model is built upon two basic principles on the relationship between the tissue-scale deformation and cell events. First, a deformed cell can recover to its original shape after the removal of external loadings [53], thus the changes in cell shape contribute to tissue elastic deformation. Second, topological transitions at the cellular scale correlate with inelastic tissue deformation [54,55]: during the cellular topological transition, neighboring cells move apart and lose their contact, while previously unconnected cells intercalate and form a new cell-cell contact (Fig 1b). This paper is organized as follows. In Model and methods, we introduce the cell-based tissue model (i.e., the vertex model) and the simulation protocol. In Section “Tissue necking instability”, we use the vertex model to simulate the uniaxial tensile test of an epithelial tissue, and reveal its necking instability at both the tissue scale and the cellular scale. In Section “Theoretical analysis”, the criteria for necking bifurcation and necking propagation are predicted based on the mean-field constitutive relation we propose, and validated by discrete vertex simulations. The effects of topological defects are discussed in Section “Effects of topological defects”. We further discuss the effects of boundary conditions in Section “Effects of boundary conditions”. We summarize the main results drawn from this study in Section “Discussion and conclusion”.

## Model and methods

### Cell-based tissue model

The mechanical behaviors of epithelial tissues can be understood from the mechanical interactions at the cellular scale, such as cell-cell adhesion and actomyosin-mediated tension along the cell edges. The vertex model is a cell-based mechanical model that links cell mechanics with tissue deformation. It treats each cell as an individual polygon containing several vertices, and each vertex represents a tri-cellular junction where cell edges meet, and on which force balance is written [35,36,43,56–60].

The potential energy of the epithelial monolayer mainly arises from the mechanical resistance of cells to their shape changes, and would build up when the actual shapes of cells deviate from their preferred shapes. Given this, the potential energy of each cell can be written as a function of cellular morphometric parameters such as cell area and cell perimeter [44,61]:


$$\begin{array}{c} E_{i}=\frac{\text{1}}{\text{2}}\left[K_{A}{\left(A_{i}-A_{\text{0}}\right)}^{\text{2}}+K_{P}{\left(P_{i}-P_{\text{0}}\right)}^{\text{2}}\right], \end{array}$$ (1)

with $A_{\text{0}}\;$(and $P_{\text{0}}$) the preferred cell area (and perimeter), $A_{i}\;$(and $P_{i}$) the actual area (and perimeter) of cell i, and $K_{A}$ (and $K_{P}$) the rigidities of cell area (and perimeter). The first energy term in Eq (1) represents the area elasticity that originates from cell cytoskeleton, while the second energy term is contributed by the line tension of the cell edges (composed of cell membrane and cortical actomyosin filaments). For epithelial tissues composed of a single type of cell, it is reasonable to assume that all the cells have the same $A_{\text{0}}\;$and $P_{\text{0}}$. Then the total energy of the epithelial tissue would be $E=\sum_{i}E_{i}$.

To simplify the numerical simulation and theoretical analysis, herein, we non-dimensionalize the cell energy by $K_{A}A_{\text{0}}^{\text{2}}$, cell area by $A_{\text{0}}$, and parameters of length (e.g., cell perimeter P and spatial coordinates) by $\sqrt{\;A_{\text{0}}}$. Then the potential energy of cell i can be non-dimensionalized as


$$\begin{array}{c} e_{i}=\;\frac{E_{i}}{K_{A}A_{\text{0}}^{\text{2}}}=\;\frac{\text{1}}{\text{2}}\left[{\left(a_{i}-\text{1}\right)}^{\text{2}}+\kappa {\left(p_{i}-\;\chi \right)}^{\text{2}}\right], \end{array}$$ (2)

with $a_{i}=\;A_{i}/A_{\text{0}}$ and $p_{i}=\frac{P_{i}}{\sqrt{\;A_{\text{0}}}}$ respectively the dimensionless area and perimeter of cell *i*. The dimensionless energy (2) is regulated by the rigidity ratio $\kappa =K_{P}/{(K}_{A}A_{\text{0}})$ and a dimensionless geometric parameter $\chi =P_{\text{0}}/\sqrt{\;A_{\text{0}}}$. The latter serves as the “shape index” of single cells [44], and has proven to be a crucial parameter that determines the initial tissue stiffness [43,44].

In the vertex model, the changes in cell shape and arrangement are achieved through the movements of polygon vertices, which are driven by the potential forces applied to them. The potential force applied to vertex α is $F_{\alpha }=-\sum_{i}\partial e_{i}/\partial r_{\alpha }$, with $r_{\alpha }$ the spatial position of vertex α. This force should be balanced with the viscous force there, that is


$$\begin{array}{c} \gamma \frac{\text{d}r_{\alpha }}{\text{d}t}=-\sum_{i}\frac{\partial e_{i}}{\partial r_{\alpha }}, \end{array}$$ (3)

with γ the viscous coefficient, whose value would not affect the quasi-static problems studied in this work. It is, however, worth noting that there are active forces and nonequilibrium effects in epithelial tissues which may introduce additional complexity [60]. Here, we did not consider such effects but note that they could be included in the model as additional forces.

Treating each cell as an infinitesimal element in a continuum (i.e., the tissue), one can also introduce stress tensors to describe the mechanical state of single cells, which are subjected to discrete point forces acting on their vertices. For instance, the relationship between Virial-like stress σ and vertex forces can be established from an identical equation σ=(r ⨂ σ)·∇. This equation holds for any second-order tensor that is symmetric and divergence-free. Integrating this equation over the cell area a and applying the divergence theorem, we have


$$\begin{array}{c} \int_{a}\sigma \text{d}a=\int_{a}(r\;⨂\;\sigma )\cdot \nabla \text{d}a=\oint_{l}r\;⨂\;\sigma \cdot n\text{d}l, \end{array}$$ (4)

where l stands for the cell boundary, and n is the unit vector normal to the cell boundary. Under the assumption of uniform stress within a cell, the mechanical state of cell i can be represented by the Virial stress $\sigma_{i}=a_{i}^{-\text{1}}\oint_{l}r_{i}\;⨂t_{i}\text{d}l$, with $t_{i}=\sigma_{i}\cdot n_{i}$ the force distributed at the cell boundary. In the vertex model, the boundary forces are discrete point forces that act on vertices associated with cell i, thus the Virial stress $\sigma_{i}$ can be estimated as


$$\begin{array}{c} \sigma_{i}=\frac{\text{1}}{a_{i}}\sum_{\beta \in \text{ cell }i}r_{\beta }⨂f_{\beta i}, \end{array}$$ (5)

with $f_{\beta i}=\partial e_{i}/\partial r_{\beta }$ the point force acting on the β-th vertex inside cell *i*. Submitting potential energy (2) into Eq (5), we obtain the specific expression of Virial stress $\sigma_{i}$ as


$$\begin{array}{c} \sigma_{i}=\left(a_{i}-\text{1}\right)I+\frac{\kappa p_{i}(p_{i}-\chi )}{a_{i}}M_{i},\; \end{array}$$ (6)

where $M_{i}$ is a symmetric cell shape tensor defined as


$$\begin{array}{c} M_{i}=\frac{\text{1}}{p_{i}}\sum_{\beta \;\in \text{ cell }i}l_{\beta ,\beta +\text{1}}p_{\beta ,\beta +\text{1}}\;{⨂\;p}_{\beta ,\beta +\text{1}}.\; \end{array}$$ (7)

Here, $p_{\beta ,\beta +\text{1}}$ is the unit vector pointing from vertex β to vertex β+1, and $l_{\beta ,\beta +\text{1}}$ is the dimensionless distance between these two vertices. See S1 File for the derivation of Eqs (6) and (7).

## Results

### Tissue necking instability

The epithelial tissue is modelled as a finite stripe tiling of uniform hexagonal cells with unit area (Fig 1c). In our simulations, the total energy of the tissue is first minimized to obtain the stress-free state, from which we perform displacement-controlled uniaxial tensile tests. The left end of the tissue stripe is fixed, while the right end is displaced along the loading direction by 0.1% of the initial tissue length at each loading step. After each loading step, the force balance Eq (3) for each vertex is solved using the forward Euler method [58,62] until a local energy minimum is reached. The time step of the simulation is initially set to dt=0.1. Convergence is defined by the absolute change in total energy between two consecutive Euler updates falling below ${\text{1}\text{0}}^{-\text{7}}$. For a given time step dt, the relaxation is allowed to proceed for up to 3000 Euler iterations. If the convergence criterion is not satisfied within these iterations, dt is reduced by a constant factor 0f 0.9 and the relaxation is continued with the smaller time step. This adaptive time-step reduction is repeated until the energy convergence criterion is satisfied, after which the simulation proceeds to the next loading step. Topological transitions are performed when an edge is shorter than 0.1 after each time step. Cells at the free boundaries are described by the same potential energy as bulk cells, and the positions of vertices at those free edges also yield the overdamped dynamic Eq (3). To examine the role of boundary line tension presented in many tissue [60,63], we also consider an additional constant boundary line tension T applied to free boundaries. Unless otherwise stated, we set the initial tissue length L=30, the initial tissue width W=10, the boundary line tension T=0, the rigidity ratio κ=0.16, and the shape index χ=3.7.

Importantly, and consistent with experimental observations (Fig 1a), the uniaxial stretching of the epithelial tissue naturally leads to necking (Fig 1c): with continued stretching, the tissue first undergoes homogeneous deformation, then locally narrows, and this narrow region gradually propagates and causes the tissue body to separate into two regions with distinct widths. In the region with a marked decrease in tissue width (i.e., the necked region), all the cells have their neighbors changed, and the cell edges along the loading direction undergo zigzag-to-armchair transition. On the other hand, cells in the less-deformed region change their shapes but not their neighbors, indicating pure elastic deformation in this region. The necked region can steadily propagate along the tissue, while the tissue width (and cell shape) in both the necked and un-necked regions remains constant (Fig 1c). During the necking propagation, the nominal tissue stress s drops to a nearly constant level (Fig 1d). Once the necked region propagates through the whole tissue, the tissue becomes homogeneous again and the tissue stress s rises up until the final catastrophic failure. The stress-stretch curve (Fig 1d) clearly shows the four stages of tissue deformation: i) initial homogeneous elastic deformation, ii) steady necking propagation after bifurcation, iii) tissue stiffening, and iv) final catastrophic failure.

**Fig 2:**
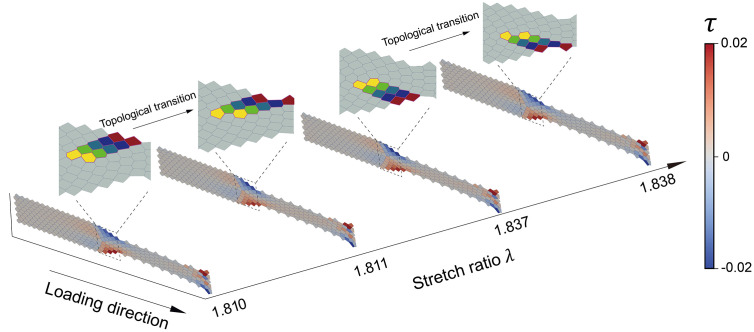
Tissue necking is accompanied by topological transitions at the cell scale. During the necking propagation, neighboring cells labeled with the same color undergo topological transitions and separate from each other. Both cellular topological transitions and shear stress τ are localized to the necking front.

The necking front of the epithelial tissue undergoes inelastic shear deformation (Fig 2), highly reminiscent of the plastic flow in metallic materials [64]. Interestingly, the tissue necking front also forms “slip lines” similar to those observed in metals. The slip lines are formed by continuous topological transition events (Fig 2): as the necked region propagates forward, the cells in front of the necked region are forced to move inward and change their positions and neighbors, and such transition events occur successively along an oblique line. Cellular topological transition rearranges the cell positions and narrows the tissue, and thus promotes the necking propagation in the epithelial tissue. Besides topological transition, the shapes of these cells are also distorted and the shear stress τ accumulates in the necking front (Fig 2).

We checked the numerical robustness of the necking simulations by varying the relaxation time step and loading increment. The resulting stress-stretch relation and necking responses remain unchanged for (dt=0.1, 0.075, 0.05) and loading increments of (0.2%, 0.1%, 0.05%) (S3 Fig).

### Theoretical analysis

As a limiting-point instability, the necking of materials is initiated when the load reaches the peak value in the uniaxial stress-stretch curve [25]. On the other hand, the steady necking propagation is analogous to “phase-separation”: the necked region and the un-necked region coexist and evolve with uniaxial stretching [21,65]. The constitutive relationship, i.e., the uniaxial stress-stretch relation of materials undergoing homogeneous deformation, has been shown to provide key information on both the necking bifurcation and the necking propagation. Therefore, in the following section, we first derive the uniaxial stress-stretch relation of epithelial tissues with homogenous deformation, then use this relation to predict the bifurcation condition for tissue necking and the tissue mechanical state during necking propagation. Comparisons between the theoretical predictions and numerical simulations are also given.

#### Mean-field constitutive model.

Consistent with the discrete vertex simulations in Model and methods, we model the tissue as a tiling of initially uniform and regular hexagonal cells. We take the initial stress-free configuration as the reference configuration, then analyze the tissue elasticity that involves changes in cell shape, and also the inelastic tissue deformation arising from topological transition at the cellular scale. The following constitutive relation is a mean-field formulation of the vertex model, rather than as a rigorous continuum limit of the vertex model [45,66]. Note that this mapping between the vertex model and continuum deformation does not necessarily satisfy material identity or continuity requirements, and is therefore a continuum analogy.

**Fig 3:**
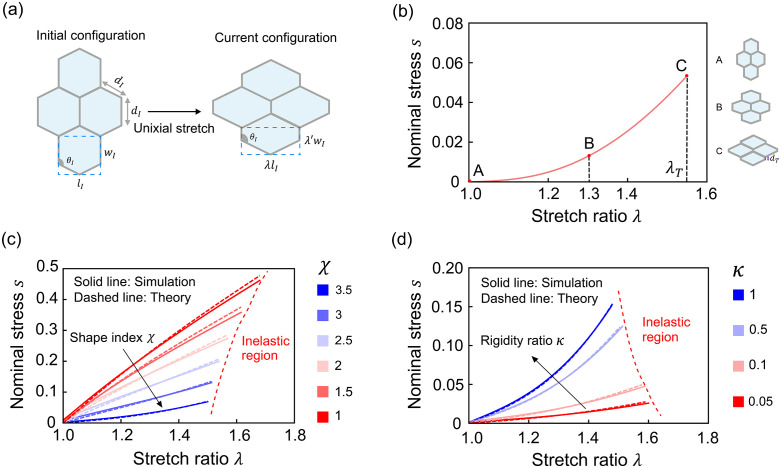
Tissue elastic deformation with changes in cell shape. **(a)** The initial and current configurations of a four-cell unit in a regular cell lattice. The left schematic defines the initial cell geometry, including the edge length $d_{I}$, interior angle $\theta_{I}$, cell length $l_{I}$ and cell width $w_{I}$. The right schematic shows the current cell length $\lambda l_{I}$ and width $\lambda^{\prime }w_{I}$, with λ and $\lambda^{\prime }$ the tissue stretch ratios. **(b)** Theoretical prediction of the tissue stress-stretch curve before topological transition (i.e., $\lambda <\lambda_{T}$, with $\lambda_{T}$ the critical stretch ratio for topological transition), shown with representative unit configurations. Discrete vertex simulations and theoretical predictions of the tissue stress-stretch curves for various (c) shape index χ and (d) rigidity ratio κ.


**Initial stress-free configuration:** In the stress-free state, cells are fully relaxed and have minimal potential energy. In each hexagonal cell, all the edges have the same length $d_{I}$ and all the interior angles are equal to ${\theta_{I}=\text{1}\text{2}}^{{\text{0}}^{\circ }}$ (Fig 3a), thus the cellular potential energy (2) can be simplified as $e_{I}\left(d_{I}\right)={\left(\frac{\text{3}\sqrt{\text{3}}}{\text{2}}{d_{I}}^{\text{2}}-\text{1}\right)}^{\text{2}}+{\kappa (\text{6}d_{I}-\chi )}^{\text{2}}$. Note that the energy $e_{I}$ becomes zero when the shape index  χ reaches the critical value $\begin{array}{c} \chi^{*}=\text{2}\sqrt{\text{6}\;\text{tan}\frac{\pi }{\text{6}}}\approx \text{3}.\text{7}\text{2} \end{array}$. In this scenario, we have the cell perimeter $p_{I}=\chi^{*}$ and the cell area $a_{I}=\text{1}$. Otherwise, we can introduce $k=\text{6}d_{I}/\chi^{*}$, which is the relative edge length with respect to the ground state. This relative length k should satisfy $\text{d}e_{I}/\text{d}d_{I}=\text{0}$, which suggests

$$\begin{array}{c} \text{2}k\left(k^{\text{2}}-\text{1}\right)+\;\chi^{*}\kappa \left(k\chi^{*}-\chi \right)=\text{0}. \end{array}$$ (8)

And we have the cell area $a_{I}$ and the cell perimeter $p_{I}$ as $a_{I}=k^{\text{2}}$ and $p_{I}=k\chi^{*}.\;$We can also characterize the initial cell shape in terms of the cell length $l_{I}$ and cell width $w_{I}$ (Fig 3a). They are related to the edge length $d_{I}$ and angle $\theta_{I}$ as$\;l_{I}=\text{2}d_{I}\text{sin}\theta_{I},\;w_{I}=d_{I}\left(\text{1}-\text{cos}\theta_{I}\right)$, and can be expressed as $l_{I}=\frac{k\chi^{*}}{\text{2}\sqrt{\text{3}}}\;$ and$w_{I}=\frac{k\chi^{*}}{\text{4}}.$

**Tissue elasticity: Cell deformation:** Subjected to external forces, the epithelial tissue will deform. Refer to the initial stress-free configuration, we can define λ and $\lambda^{\prime }$ as the tissue stretch ratios respectively along the length and width directions. In the absence of cell rearrangement, cell shape changes are synchronized with the tissue deformation, which means the current cell length and width respectively become $\lambda l_{I}$ and $\lambda^{\prime }w_{I}$ (Fig 3a), and the current cell area $a=\lambda \lambda^{\prime }k^{\text{2}}$ and perimeter $p=\left[\frac{f(\theta )\lambda }{\sqrt{\text{3}}}+\lambda^{\prime }\right]\frac{k\chi^{*}}{\text{2}}$, with f(θ)=(2+cosθ)/sinθ. Submitting a and p into Eq (2), we can obtain the potential energy of a single cell as


$$\begin{array}{c} {e(\lambda ,\;\lambda^{\prime },\theta )=\frac{\text{1}}{\text{2}}\left(\lambda \lambda^{\prime }k^{\text{2}}-\text{1}\right)}^{\text{2}}+\frac{\kappa }{\text{2}}{\left\{\left[\frac{f(\theta )\lambda }{\sqrt{\text{3}}}+\lambda^{\prime }\right]\frac{k\chi^{*}}{\text{2}}-\chi \right\}}^{\text{2}}. \end{array}$$ (9)

Besides two stretch ratios λ and $\lambda^{\prime }$, the energy (9) is also dependent on the interior angle θ. In a regular cell lattice, the tissue energy density equals the energy of a single cell divided by its initial area $a_{I}$, that is $e/k^{\text{2}}$. Then, the nominal tissue stresses can be written as


$$\begin{array}{c} \;s=\;\frac{\partial e(\lambda ,\;\lambda^{\prime },\theta )}{k^{\text{2}}\partial \lambda },\;s^{\prime }=\;\frac{\partial e(\lambda ,\;\lambda^{\prime },\theta )}{k^{\text{2}}\partial \lambda^{\prime }}.\; \end{array}$$ (10)

As the angle θ can adjust freely, the energy minimization requires $\frac{\partial e(\lambda ,\;\lambda^{\prime },\theta )}{\partial \theta }=\text{0},$ which suggests $f^{\prime }(\theta )=\text{0}$, that is $\theta ={\text{1}\text{2}}^{{\text{0}}^{\circ }}$. This means epithelial cells always undergo isogonal deformation [67]. Therefore, the nominal stresses (10) yield


$$\begin{array}{c} \;s=\lambda^{\prime }\left(k^{\text{2}}\lambda \lambda^{\prime }-\text{1}\right)+\frac{\kappa \chi^{*}\left[-\text{2}\chi +k\chi^{*}(\lambda +\lambda^{\prime })\right]}{\text{4}k}, \end{array}$$ (11)

$$\begin{array}{c} s^{\prime }=\lambda \left(k^{\text{2}}\lambda \lambda^{\prime }-\text{1}\right)+\frac{\kappa \chi^{*}\left[-\text{2}\chi +k\chi^{*}(\lambda +\lambda^{\prime })\right]}{\text{4}k}. \end{array}$$ (12)

For the uniaxial tensile test (Fig 1c and 1d), the transverse stress $s^{\prime }=\text{0}$ (12) and the longitudinal nominal stress s can be determined as (Fig 3b)


$$\begin{array}{c} s=\frac{\text{2}\kappa \chi^{*}\left(\chi^{*}-\text{2}k\chi \lambda +k^{\text{2}}\chi^{*}\lambda^{\text{2}}\right)[\kappa \chi^{*}\left(-\chi +k\chi^{*}\lambda \right)+\text{2}k\lambda \left(-\text{1}+k^{\text{2}}\lambda^{\text{2}}\right)]}{{k\left(\kappa {\chi^{*}}^{\text{2}}+\text{4}k^{\text{2}}\lambda^{\text{2}}\right)}^{\text{2}}}. \end{array}$$ (13)

In discrete vertex simulations (Fig 1d), we have the nominal stress s= $\sigma \lambda^{\prime }$, with σ the uniaxial Virial stress calculated from Eq (6). The theoretical prediction of the tissue stress (13) fits well the simulation results in various parameter settings (Fig 3c and 3d). We also find that the shape index χ and the rigidity ratio κ show opposite effects on tissue stiffness: when the shape index χ increases and gets closer to the critical value $\chi^{*}$, the single cells have a smaller potential energy (see Subsection “Initial stress-free configuration” for details) and thus become easier to deform; while an increase in the rigidity ratio κ would increase the line tension at the cell edges, raise the cellular potential energy (9), and thereby stiffen the cells.

**Fig 4:**
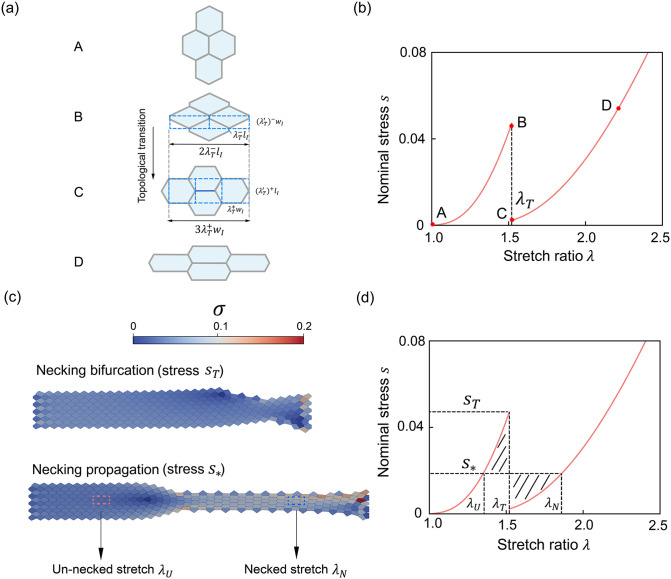
Tissue inelastic deformation and necking predictions. **(a)** Schematics of a four-cell unit that undergoes topological transition. At the transition, the overall length of the cell unit remains constant, but the cell shape changes abruptly: the longitudinal cell stretch jumps from $\lambda_{T}^{-}$ to $\lambda_{T}^{+}$, while the transverse stretch jumps from ${\left(\lambda_{T}^{\prime }\right)}^{-}$ to ${\left(\lambda_{T}^{\prime }\right)}^{+}$. **(b)** Theoretical stress-stretch relation of an epithelial tissue with homogeneous deformations, where tissue inelasticity arises from cellular topological transition that occurs at ${\lambda =\lambda }_{T}$. **(c)** Simulation snapshots for (upper) necking bifurcation and (lower) necking propagation. The second snapshot shows the coexistence of the necked region with stretch $\lambda_{N}$ and the un-necked region with stretch $\lambda_{U}$. **(d)** The theoretical stress-stretch relation (for homogeneously deformed tissues) reveals the bifurcation stress $s_{T}$ and the mechanical state during necking propagation, characterized by the propagation stress $s_{*}$ and two stretch ratios x$\lambda_{U}$ and $\lambda_{N}$. The shaded regions have equal areas.


**Tissue inelasticity: cell topological transition:** Next, we examine the tissue inelastic deformation associated with topological transition (i.e., cell rearrangement) (Fig 4a). During stretching, cell edges perpendicular to the loading direction progressively shrink, and once their length falls below a critical threshold, new edges form along the loading direction, leading to cell rearrangement. We denote $\lambda_{T}$ as the critical stretch ratio when the topological transition occurs. It should satisfy the critical condition $d=d_{T}\;$(set as 0.1 in this study), that is

$$\begin{array}{c} \chi^{*}\left[\text{6}k\lambda_{T}-\text{2}k^{\text{3}}{\lambda_{T}}^{\text{3}}+\kappa \chi^{*}\left(\text{3}\chi -\text{2}k\chi^{*}\lambda_{T}\right)\right]=\text{6}\left(\kappa {\chi^{*}}^{\text{2}}+\text{4}k^{\text{2}}{\lambda_{T}}^{\text{2}}\right){\;d}_{T}. \end{array}$$ (14)

At the transition point, the tissue stretch ratio $\lambda_{T}$ remains continuous, but the cellular stretch undergoes a discrete change due to rearrangement. Let $\lambda_{T}^{-}$ and $\lambda_{T}^{+}$ respectively be the cellular stretch ratios right before and after the transition point. Since the tissue deformation is purely elastic before transition, we immediately have $\lambda_{T}^{-}=\lambda_{T}$. After the topological transition, however, cells change positions and the cellular stretch ratio $\lambda_{T}^{+}$ differs from the tissue stretch ratio $\lambda_{T}$. Given that the total length of a four-cell unit remains constant at transition (see schematics B and C in Fig 4a) and this cell unit now has three cells instead of two along the length direction, we can establish the relationship between two cellular stretch ratios as $\text{2}\lambda_{T}^{-}l_{I}=\text{3}\lambda_{T}^{+}w_{I}$, which suggests


$$\begin{array}{c} \lambda_{T}^{+}=n\lambda_{T},\text{ with }n=\frac{\text{4}\sqrt{\text{3}}}{\text{9}}. \end{array}$$ (15)

One can easily find $\lambda_{T}^{+}<\lambda_{T}^{-}=\lambda_{T}$, indicating that the topological transition mitigates cellular shape changes by redistributing the tissue-level elongation among more cells along the load-bearing axis. As a result, both the stretch and the stress carried by each cell are reduced.

Further tissue deformation after the topological transition still arises from changes in cell shape. Since the current tissue length is stretched by a factor $\lambda /\lambda_{T}$ relative to the transition point, the corresponding cellular stretch becomes $\lambda_{T}^{+}\left(\lambda /\lambda_{T}\right)=n\lambda$. Following Subsection “Tissue elasticity: Cell deformation”, we obtain the tissue energy density $e(\lambda ,\lambda^{\prime },\theta )$ and corresponding nominal stress s(λ) with$\;\lambda >\lambda_{\text{T}}$ (see S1 File for details). The complete stress-stretch relation including topological transition can be summarized as


$$\begin{array}{c} s=\left\{ \begin{array}{c} \frac{\text{2}\kappa \chi^{*}\left(\chi^{*}-\text{2}k\chi \;\lambda +k^{\text{2}}\chi^{*}{\;\lambda }^{\text{2}}\right)\left[\kappa \chi^{*}\left(-\chi +k\chi^{*}\;\lambda \right)+\text{2}k\;\lambda \left(-\text{1}+k^{\text{2}}{\;\lambda }^{\text{2}}\right)\right]}{{k\left(\kappa {\chi^{*}}^{\text{2}}+\text{4}k^{\text{2}}{\;\lambda }^{\text{2}}\right)}^{\text{2}}}\;\lambda <\lambda_{\text{T}}\;\\ \frac{\text{2}\kappa \chi^{*}\left[\chi^{*}-\text{2}k\chi n\lambda +k^{\text{2}}\chi^{*}({n\lambda )}^{\text{2}}\right]\left\{\kappa \chi^{*}\left(-\chi +k\chi^{*}n\lambda \right)+\text{2}kn\lambda \left[-\text{1}+k^{\text{2}}({n\lambda )}^{\text{2}}\right]\right\}}{\sqrt{\text{3}}k{\left[\kappa \chi^{*}+\text{4}k^{\text{2}}{\left(n\lambda \right)}^{\text{2}}\right]}^{\text{2}}}\;\lambda >\lambda_{\text{T}} \end{array}\right.\;. \end{array}$$ (16)

This stress-stretch relation is discontinuous as the topological transition causes stress relaxation of the tissue (Fig 4b). Note that although topological transitions relax cell-level stretch and contribute to the inelastic component of tissue deformation, they should be viewed as discrete, non-local events rather than constitutive strain as defined in continuum mechanics.

#### Analysis of tissue necking.

The necking bifurcation occurs when the tissue stress reaches its peak value [21,25]. From the discontinuous constitutive relation (16), the necking bifurcation is coincident with the topological transition. This means the tissue stretch ratio at bifurcation is just $\lambda_{T}$ in Eq (14) and the bifurcation stress is $s_{T}=s(\lambda_{T})$ in Eq (13).

After the onset of necking, the tissue will enter the stage where the necked phase steadily propagates forwards (Fig 1c). In this stage, the nominal stress remains constant at a value $s_{*}$ (Fig 1d). To analyze this process, we consider an infinitesimal tissue section (with initial length dL) in front of the neck. When the neck front shifts forward to engulf this section, this section undergoes significant deformation with a length increase of $\left(\lambda_{\text{N}}-\lambda_{\text{U}}\right)\text{d}L$, where $\lambda_{\text{N}}$ and $\lambda_{\text{U}}$ are respectively the stretch ratios in the necked and un-necked states (Fig 4c). The work done is therefore $s_{*}(\lambda_{\text{N}}-\lambda_{\text{U}})\text{d}L$. This work should be equal to $\text{d}W=\text{d}L\int_{\lambda_{\text{U}}}^{\lambda_{\text{N}}}s(\lambda )\text{d}\lambda$, which is the energy increase of this section as it passes from the un-necked state to the necked state. Then the propagation stress $s_{*}$ should yield


$$\begin{array}{c} s_{*}\left(\lambda_{\text{N}}-\lambda_{\text{U}}\right)=\int_{\lambda_{\text{U}}}^{\lambda_{\text{N}}}s(\lambda )\text{d}\lambda =\int_{\lambda_{\text{U}}}^{\lambda_{\text{T}}}s(\lambda )\text{d}\lambda +\int_{\lambda_{\text{T}}}^{\lambda_{\text{N}}}s(\lambda )\text{d}\lambda ,\; \end{array}$$ (17)

where s(λ) is given in Eq (16). Eq (17) has a simple graphical explanation (Fig 4d): the rectangular area $s_{*}\left(\lambda_{\text{N}}-\lambda_{\text{U}}\right)$ is equal to the area under the stress-stretch curve s(λ) in the interval from $\lambda_{\text{U}}$ to $\lambda_{\text{N}}$, thus the areas of the two lobes (i.e., the shaded regions in Fig 4d) are also equal. This equality is also known as Maxwell’s condition for the coexistence of two phases [65,68]. Once the propagation stress $s_{*}$ is determined, one can also obtain the stretch ratios $\lambda_{\text{U}}$ and $\lambda_{\text{N}}$ from the stress-stretch relation (17) (Fig 4d).

**Fig 5:**
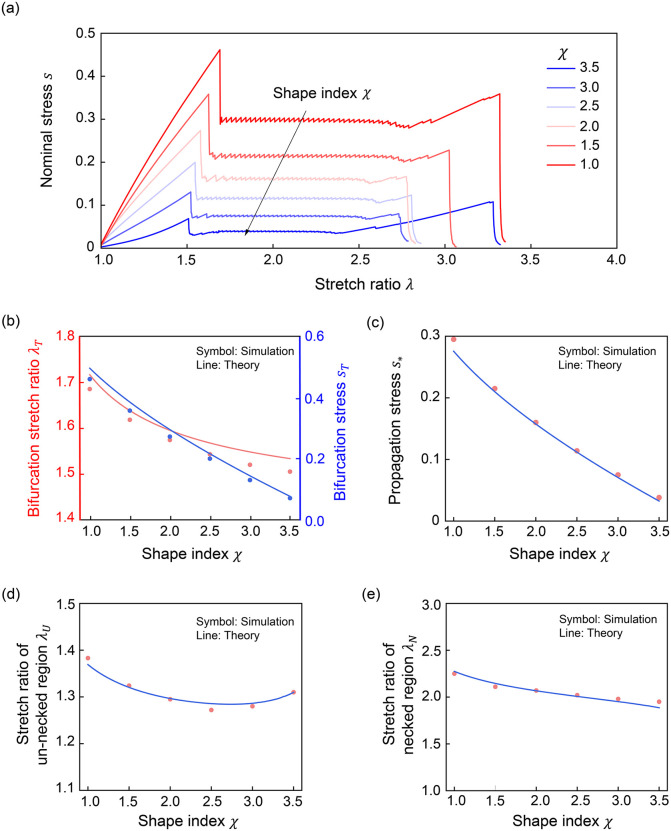
Theoretical predictions and vertex simulations of tissue necking with various shape indices χ. **(a)** Simulation results of the uniaxial stress-stretch curves for different values of χ. Comparison of theoretical predictions with simulation results for the (b) bifurcation stress $s_{T}$ and corresponding stretch ratio $\lambda_{T}$, (c) propagation stress $s_{*}$, (d) stretch ratio of the un-necked region $\lambda_{U}$, and (e) stretch ratio of the necked region $\lambda_{N}$.

Our theoretical predictions fit well the simulation results in various parameter settings, and reveal the dependence of tissue necking behaviors on tissue properties (such as the shape index χ) (Fig 5a). As mentioned in Subsection “Tissue elasticity: Cell deformation”, an epithelial tissue with a larger shape index χ is mechanically softer, thus the bifurcation stress $s_{T}$ and propagation stress $s_{*}$ also become smaller with the increase in χ (Fig 5b and 5c). On the other hand, the tissue stretch ratios, represented by $\lambda_{\text{N}}$ (for necked region) and $\lambda_{\text{U}}$ (for un-necked region), are insensitive to the shape index χ (Fig 5d and 5e). Moreover, changing the topological transition threshold $d_{T}$ shifts the necking behaviors in a systematic way: a smaller threshold gives a larger bifurcation stress $s_{T}$ and propagation stress $s_{*}$, consistent with our theoretical prediction (S4 Fig).

**Fig 6:**
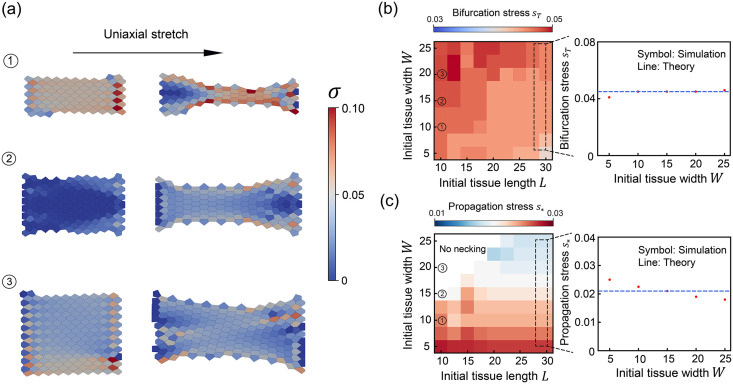
Influence of tissue dimensions on tissue necking. **(a)** Simulation snapshots of epithelial tissues with different initial widths W (with the initial length L=10). Stretch ratios in the first and second snapshots are respectively 1.4, 2.1. Phase diagrams illustrating the influence of tissue length L and width W on the (b) bifurcation stress $s_{T}$ and (c) propagation stress $s_{*}$. As the tissue width W increases (with L=30), both the bifurcation stress$\;s_{T}$ and propagation stress $s_{*}\;$remain close to theoretical predictions.

In our theoretical analysis based on the uniaxial stress-stretch relation, the bifurcation condition is independent of the initial tissue length or width, which is also consistent with the vertex simulation results (Fig 6a and 6b): the bifurcation stress $s_{T}$ remains close to the theoretical prediction when the tissue length or width varies. We notice that, as the initial tissue width W increases, the bifurcation transforms from a symmetric mode to an anti-symmetric mode (Fig 6a), the latter leads to localized shearing instead of necking [69]. Fig 6c shows that the symmetric bifurcation and corresponding necking instability always occur unless the initial tissue width W becomes larger than the tissue length L, and the values of the propagation stress $s_{*}$ obtained from vertex simulations consistently align with the theoretical prediction (17). Overall, the validation of the theoretical predictions demonstrates that our theoretical model efficiently captures the general features of the necking of epithelial tissues.

### Effects of topological defects

In previous sections, epithelial cells are assumed to be well-organized into regular hexagonal shapes. This assumption enables to derive simple constitutive descriptions of the epithelial tissue, and yields key predictions regarding tissue necking. In real epithelial tissues, however, cell shapes can be irregular and the cell arrangement is also disordered [43,55,70], see also Fig 1b for the epithelium of a *Drosophila* embryo [71]. A notable feature is the presence of epithelial cells with fewer or more than six edges, resembling pentagons or heptagons rather than hexagons. Typically, a pentagonal cell pairs with a heptagonal one, forming a pentagon-heptagon defect pair [72–74]. Analogous to two-dimensional carbon materials like graphene [75], these non-hexagonal cells can act as topological defects and alter tissue deformation [58,76]. Given these, we further investigate the influence of topological defects on tissue necking by performing vertex simulations. We find that, in the elastic deformation stage, the nonlinear stress-stretch curves of tissues with topological defects closely match those of ordered tissues (i.e., hexagonal cell lattice) (S1 Fig), indicating that tissue elasticity is insensitive to topological defects. The inelastic tissue deformation and the tissue necking behavior, however, can be affected by topological defects, as detailed below.

**Fig 7:**
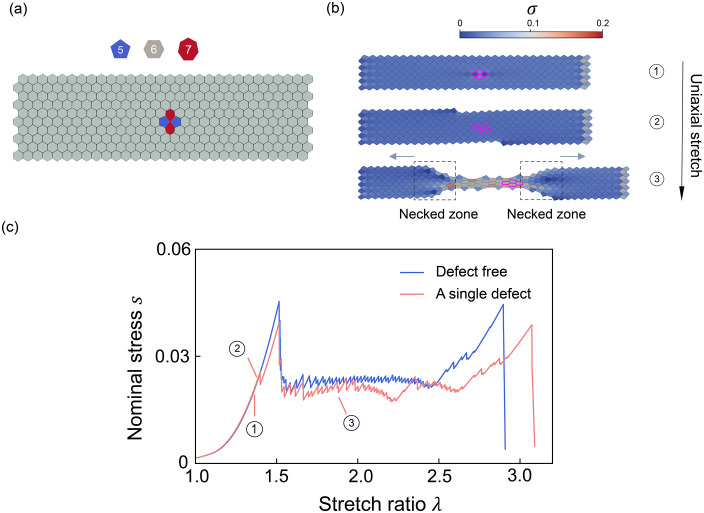
Necking behavior of the epithelial tissue with a pentagon-heptagon defect pair. **(a)** The initial tissue configuration and **(b)** deformed configurations, showing that necking initiates from the defect site. **(c)** Corresponding uniaxial stress-stretch response, compared to that of a defect-free tissue (see also Fig 1d).

To evaluate the impact of topological defects on tissue necking, we first embed a pentagon-heptagon pair into a regular cell lattice to form a dispersed “point defect” (Fig 7a). Such topological pairs are widely observed in epithelial tissues across diverse species [73]. We find that even a single point defect is sufficient to trigger the tissue neck to initiate from the site where the defect is located (Fig 7b), indicating that the necking bifurcation of tissues is highly sensitive to topological defects. Such high sensitivity to material imperfections is also a characteristic of the necking of metallic materials [77]. In contrast, this defect pair has little impact on the overall stress-stretch curve (Fig 7c).

**Fig 8:**
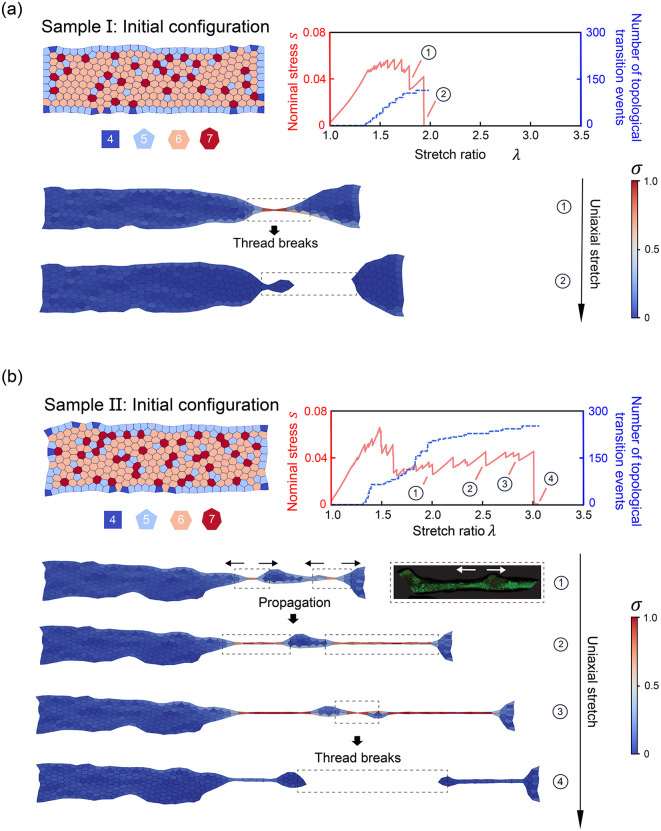
Necking behaviors of disordered tissues. Disordered tissues exhibit two necking modes: **(a)** catastrophic failure without necking propagation; **(b)** failure preceded by persistent necking propagation. For each mode, the panels display a representative initial configuration alongside the evolution of the nominal stress s and the cumulative number of topological transition events as a function of the tissue stretch ratio λ. The shape index is χ=3.5 in simulations.

Next, we generate disordered cell lattices using Voronoi tessellation [35,60,78]. The randomness of topological defects leads to diverse necking behaviors across different tissue samples. The number of topological transition events (blue dotted line in Fig 8) increases after the bifurcation and is synchronized with the fluctuations of the stress curve (red line in Fig 8), where pronounced stress drops occur due to cell rearrangements. Following the necking bifurcation, some tissue samples undergo catastrophic failure directly, without necking propagation (e.g., Sample I in Fig 8a), whereas in others (e.g., Sample II in Fig 8b), the neck region propagates persistently, as in ordered tissues (i.e., regular cell lattice in Fig 1c and 1d). Apparently, the second mode exhibits higher ductility and toughness than the first one. Both necking modes can be observed in tissue samples with diverse shapes and sizes (see S2 Fig for details, where the tissue aspect ratio L/W varies from 1 to 4 and the total cell number N=150, 300, 600), and in both cases, the necked region collapses into a thin thread before final rupture, reminiscent of the morphological evolution of *Trichoplax adhaerens* (Fig 1a). In the second necking mode, the disordered tissue may develop multiple necked regions that propagate towards each other, a phenomenon also observed in experiments (Fig 8b).

**Fig 9:**
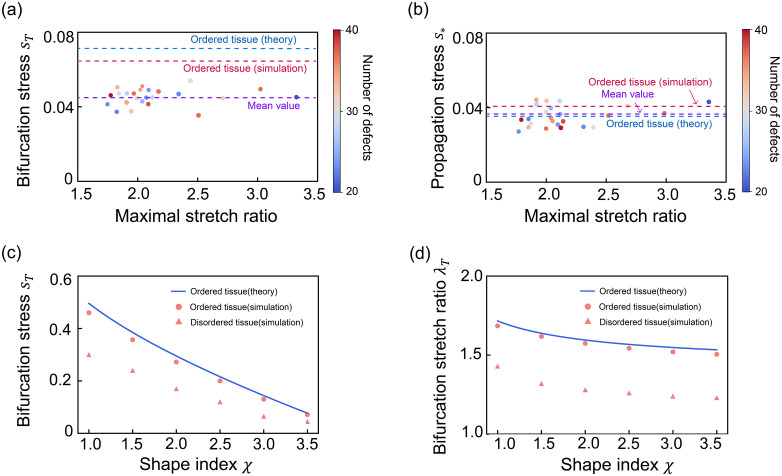
Necking stresses of disordered tissues. **(a)** The bifurcation stress $s_{T}$ and **(b)** propagation stress $s_{*}$ of disordered tissues with random topological defects, where the symbol color represents the number of initial defects and the mean stress values (dashed lines) are compared with those of the ordered (defect-free) tissues. Dependence of the **(c)** bifurcation stress $s_{T}$ and **(d)** bifurcation stretch $\lambda_{T}\;$on the shape index χ for both disordered and ordered tissues.

The bifurcation stress $s_{T}$ remains consistent across disordered tissue samples, albeit at a lower level than in ordered tissues (Fig 9a). This indicates that initial defects facilitate necking bifurcation. Although the occurrence and extent of necking propagation can be greatly affected by topological defects (Figs 7 and 8), the necking propagation stress $s_{*}$ across disordered samples remains close to that of ordered tissues (Fig 9b). The maximum stretch ratio, defined as the stretch at which the tissue undergoes complete failure, varies across samples; however, for most tissues it is clustered around a value of approximately 2.0. Finally, we find that necking bifurcation in disordered tissues is governed by tissue properties in a manner analogous to ordered tissues (Fig 9c and 9d). This suggests that our parameter analysis on ordered tissues (see section Theoretical analysis for details) provides a qualitative framework applicable to disordered systems.

### Effects of boundary conditions

**Fig 10:**
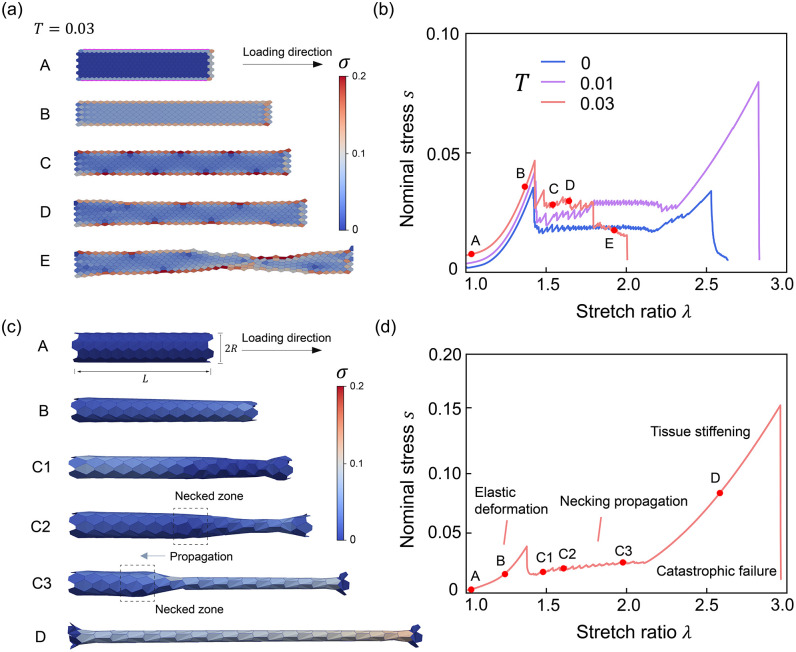
Influence of boundary conditions and geometry on tissue necking. **(a)** Representative snapshots of stripe tissues with boundary line tension T=0.03 at different stages; the free boundaries subject to line tension are highlighted in pink at stage **A. (b)** Corresponding nominal stress-stretch curves for different values of T. **(c)** Representative snapshots of a cylindrical tissue under uniaxial tension at different stages: (A) initial stress-free state, (B) elastic deformation, (C1–C3) necking bifurcation and propagation, and (D) tissue stiffening. The spatial distributions of the uniaxial stress are shown in each snapshot. **(d)** The resulting nonlinear stress-stretch response of the cylindrical tissue under uniaxial tension.

To examine whether the observed necking behavior in section “Tissue necking instability” is sensitive to boundary conditions, we first vary the line tension T applied to free boundaries. As shown in Fig 10a and 10b, increasing T modifies the stress distribution near free boundaries and smooth the tissue boundaries. A moderate boundary tension (T=0.01) increases the overall stress level, while the deformation remains essentially unchanged: the tissue still undergoes an initial stage of homogeneous deformation, followed by steady necking propagation and final failure. In contrast, at a higher boundary tension (T=0.03), although the bifurcation threshold remains nearly unchanged, the initial topological transitions occur more uniformly in the tissue instead of occurring at the boundary edge, which suppresses neck localization and inhibits necking propagation. As a result, the tissue fails at a smaller stretch. These results show that boundary line tension can alter the quantitative mechanical response and the post-bifurcation dynamics, while the onset of necking bifurcation remains robust.

We further examine a cylindrical geometry to assess whether the same mechanism persists beyond finite planar stripes. As shown in Fig 10c and 10d, the tissue again exhibits the same four stages of deformation observed in the stripe geometry. This indicates that the mechanism identified in this work may apply not only to stripe-like tissues, but also to duct-like epithelial tissues under extensive pressure, which are commonly found in development and homeostasis [79,80]. Together, these results show that the necking mechanism is robust across diverse boundaries and configurations of epithelial tissues.

### Discussion and conclusion

In this article, we integrate discrete vertex simulations with theoretical modeling to unravel the necking instability of epithelial tissues. We propose an analytical constitutive model that links cell-scale mechanical events to tissue-scale necking behavior. Specifically, the cellular shape evolution accounts for the elastic part of the tissue response, whereas topological transitions are represented in our mean-field description as discrete rearrangement events associated with stress relaxation and the inelastic component of deformation. Based on this constitutive model, we predict the bifurcation condition for tissue necking and the steady state of necking propagation. Vertex simulations quantitatively validate our theoretical predictions. We further extend our tissue model from ordered cell lattices to disordered ones, and find that initial topological defects influence the necking bifurcation and impede the necking propagation. Our simulations of disordered tissues recapitulate the necking morphologies of real epithelial tissues. We also examine the effects of boundary conditions, and find that the necking behavior is robust in moderate tissue surface tension and cylindrical geometry. Beyond necking, our work also provides valuable insights for understanding other large-scale deformation behaviors of epithelial tissues, such as the gastrulation [81] and body axis elongation [71] of embryos, and the morphogenesis of skin cancers [82], although active forces and the non-equilibrium nature of these processes may add complexity [60,83].

Additional mechanical characteristics of epithelial cells can also be included in the model to more accurately recapitulate the large-scale tissue deformations observed *in vivo*. For instance, it has been revealed that cells can actively adjust their shapes, and even push and pull on one another to create internal forces that trigger large-scale deformation [50,60,84,85]. In addition, single cells [86] have been reported to exhibit complex viscoelastic transition from solid-like to fluid-like state depending on the driving frequencies. Moreover, fundamental physiological processes such as cell division and apoptosis have been recognized to affect the tissue mechanical properties and drive tissue morphogenesis [87–90]. In that scenario, viscous dissipation arising from cell-cell interactions [91] may become important and lead to different deformation and failure behaviors [92]. Besides, large deformations of tissues ultimately lead to rupture [93], signifying the failure of cell-cell contacts. Recent evidence shows the importance of junctional mechanics and viscous dissipation in this process [93]. To better capture tissue rupture, our constitutive description effective constitutive description could in the future be refined to include details on the fracturing of cell-cell junctions [58]. Addressing these issues will require the development of refined theories in tissue mechanics.

## Supporting information

S1 Fig Comparison of elastic stress-stretch curves between ordered and disordered tissues. (DOCX)

S2 FigTwo necking modes observed in disordered tissues across a range of geometric parameters.**(a)** Various aspect ratios L/W with a fixed cell number N=150. **(b)** Various cell numbers N with L/W=3. The shape index is χ=3.5 in simulations.(DOCX)

S3 FigSimulation results with different time steps and loading increments.The uniaxial stress-stretch curves for different **(a)** time steps and **(b)** loading increments. The shape index is χ=3.5. These results confirm that a time step of dt = 0.1 and a loading increment of 0.1% provide sufficient accuracy, thus these parameter values are adopted for all simulations.(DOCX)

S4 FigInfluence of topological transition threshold on tissue necking.**(a)** Simulation results of the uniaxial stress-stretch curves for different values of topological transition threshold $d_{T}$. Comparison of theoretical predictions with simulation results for **(b)** bifurcation stress $s_{T}\;$and corresponding stretch ratio $\lambda_{T}$, **(c)** propagation stress $s_{*}.$ The shape index is χ=3.5.(DOCX)

S1 File Supporting information file. (DOCX)
