# Supplementary material for: Necking of epithelial tissues with cellular topological transition
Source: PLoS Comput Biol. 2026 Jul 29;22(7):e1014565. doi: 10.1371/journal.pcbi.1014565 (PMC13436786; doi:10.1371/journal.pcbi.1014565)
Supplement: Supplementary file 2 [file pcbi.1014565.s002.docx]

**S2 Fig.** **Two necking modes observed in disordered tissues across a range of geometric parameters.** (a) Various aspect ratios $L/W$ with a fixed cell number $N=150$. (b) Various cell numbers $N$ with $L/W=3$. The shape index is $\chi=3.5$ in simulations.
