# Supplementary material for: Necking of epithelial tissues with cellular topological transition
Source: PLoS Comput Biol. 2026 Jul 29;22(7):e1014565. doi: 10.1371/journal.pcbi.1014565 (PMC13436786; doi:10.1371/journal.pcbi.1014565)
Supplement: Supplementary file 3 [file pcbi.1014565.s003.docx]

**S3 Fig. Simulation results with different time steps and loading increments.** The uniaxial stress-stretch curves for different (a) time steps and (b) loading increments. The shape index is $\chi=3.5$. These results confirm that a time step of $dt = 0.1$ and a loading increment of 0.1% provide sufficient accuracy, thus these parameter values are adopted for all simulations.
