# Supplementary material for: Necking of epithelial tissues with cellular topological transition
Source: PLoS Comput Biol. 2026 Jul 29;22(7):e1014565. doi: 10.1371/journal.pcbi.1014565 (PMC13436786; doi:10.1371/journal.pcbi.1014565)
Supplement: Supplementary file 4 [file pcbi.1014565.s004.docx]

**S4 Fig. Influence of topological transition threshold on tissue necking.** (a) Simulation results of the uniaxial stress-stretch curves for different values of topological transition threshold $d_{T}.$ Comparison of theoretical predictions with simulation results for (b) bifurcation stress $s_{T}$and corresponding stretch ratio $\lambda_{T}$, (c) propagation stress $s_{*}.$ The shape index is $\chi=3.5$.
