# Supplementary material for: Necking of epithelial tissues with cellular topological transition
Source: PLoS Comput Biol. 2026 Jul 29;22(7):e1014565. doi: 10.1371/journal.pcbi.1014565 (PMC13436786; doi:10.1371/journal.pcbi.1014565)
Supplement: Supplementary file 5 [file pcbi.1014565.s005.docx]

**1	Cauchy stress in the vertex model**

In this section, we derive the expression of Cauchy stress in the vertex model. From Eq (5) in the main text, the Cauchy stress of cell $i$ $\boldsymbol{\sigma}_{i}$ is dependent on the discrete point force $\boldsymbol{f}_{\beta i}=\partial e_{i}/\partial\boldsymbol{r}_{\beta}$, which acts on the $\beta$-th vertex inside cell *i*. Here, the cellular potential energy $e_{i}$ is a function of cell area $a_{i}$ and cell perimeter $p_{i}$, that is $e_{i}=e_{i}(a_{i},p_{i})$ (see Eq (2) in the main text). Thus, the point force $\boldsymbol{f}_{\beta i}$ can be expanded as

$$\boldsymbol{f}_{\beta i}=\frac{\partial e_{i}}{\partial a_{i}}\frac{\partial a_{i}}{\partial\boldsymbol{r}_{\beta}}+\frac{\partial e_{i}}{\partial p_{i}}\frac{\partial p_{i}}{\partial\boldsymbol{r}_{\beta}}, (S1)$$

where the derivatives of cell area $a_{i}$ and cell perimeter $p_{i}$ with respect to $\boldsymbol{r}_{\alpha}$are given as

$$\frac{\partial a_{i}}{\partial\boldsymbol{r}_{\beta}}=\frac{1}{2}\boldsymbol{k\times}\left( \boldsymbol{r}_{\beta-1}\boldsymbol{-}\boldsymbol{r}_{\beta+1} \right)\boldsymbol{,}\frac{\partial p_{i}}{\partial\boldsymbol{r}_{\beta}}\boldsymbol{=}\frac{\boldsymbol{r}_{\beta}\boldsymbol{-}\boldsymbol{r}_{\beta-1}}{\left| \boldsymbol{r}_{\beta}\boldsymbol{-}\boldsymbol{r}_{\beta-1} \right|}\boldsymbol{+}\frac{\boldsymbol{r}_{\beta}\boldsymbol{-}\boldsymbol{r}_{\beta+1}}{\left| \boldsymbol{r}_{\beta}\boldsymbol{-}\boldsymbol{r}_{\beta+1} \right|}\boldsymbol{.}(S2)$$

In Eq (S2), $\boldsymbol{k}$ denotes the unit vector normal to the epithelial monolayer; $\boldsymbol{r}_{\beta-1}$ and $\boldsymbol{r}_{\beta+1}$ are two vertices next to the vertex $\beta$ within cell *i*. Considering the specific expression of the potential energy $e_{i}$, we have

$$\frac{\partial e_{i}}{\partial a_{i}} =(a_{i}-1)\boldsymbol{,}\frac{\partial e_{i}}{\partial p_{i}} =\kappa(p_{i}-\chi)\boldsymbol{.}(S3)$$

Submitting Eq (S2) and (S3) into (S1), we can rewrite $\boldsymbol{f}_{\beta i}$ as

$$\boldsymbol{f}_{\beta i}=\frac{1}{2}\left( a_{i}-1 \right)\left[ \boldsymbol{k\times}\left( \boldsymbol{r}_{\beta-1}\boldsymbol{-}\boldsymbol{r}_{\beta+1} \right) \right]\boldsymbol{+}\kappa\left( p_{i}-\chi\right)\left( \frac{\boldsymbol{r}_{\beta}\boldsymbol{-}\boldsymbol{r}_{\beta-1}}{\left| \boldsymbol{r}_{\beta}\boldsymbol{-}\boldsymbol{r}_{\beta-1} \right|}\boldsymbol{+}\frac{\boldsymbol{r}_{\beta}\boldsymbol{-}\boldsymbol{r}_{\beta+1}}{\left| \boldsymbol{r}_{\beta}\boldsymbol{-}\boldsymbol{r}_{\beta+1} \right|} \right) (S4)$$

Submitting Eq (S4) into the general expression of Cauchy stress $\boldsymbol{\sigma}_{i}$ in Eq (5), we obtain

$$\begin{aligned} \boldsymbol{\sigma}_{i}=\left( a_{i}-1 \right)\boldsymbol{I+}\frac{\kappa p_{i}\left( p_{i}-\chi\right)}{a_{i}}\boldsymbol{M}_{i}\boldsymbol{,}\#(S5) \end{aligned}$$

where $\boldsymbol{M}_{i}$ is a symmetric cell shape tensor defined as

$$\begin{aligned} \boldsymbol{M}_{i}\boldsymbol{=}\frac{1}{p_{i}}\sum_{\beta\in\mathrm{ce}ll i} l_{\beta,\beta+1}\boldsymbol{p}_{\beta,\beta+1} {\bigotimes\boldsymbol{p}}_{\beta,\beta+1}.\boldsymbol{\#(}S6) \end{aligned}$$

Here, $\boldsymbol{p}_{\beta,\beta+1}$ is the unit vector pointing from vertex $\beta$ to vertex $\beta+1$, and $l_{\beta,\beta+1}=\left\lfloor\boldsymbol{r}_{\beta}\boldsymbol{-}\boldsymbol{r}_{\beta+1} \right\rfloor$ is the distance between these two vertices.

**2	Stress-stretch relation after topological transition**

Here, we analyze the elastic mechanical response of epithelial tissues after topological transition (i.e. the tissue stretch ratio $\lambda>\lambda_{T}$), in a way similar to the analysis in Subsection 4.1.1. After the topological transition, cells rearrange and the four-cell unit shows a new configuration (see schematics C and D in Fig 5a). Let $\lambda_{c}$ and $\lambda_{c}^{'}$ be the cellular stretch ratios respectively along the length and width directions. Then the deformed cell has length $\lambda_{c}w_{I}$ and width $\lambda_{c}^{'}l_{I}$, with $l_{I}$ and $w_{I}$ the initial cell length and width defined in Eq (4.3), and its area $a$ and perimeter $p$ become

$$\begin{aligned} a=\lambda_{c}\lambda_{c}^{'}a_{I}=\lambda_{c}\lambda_{c}^{'}k^{2}, \\ p=2\lambda_{c}w_{I}+f\left( \theta\right)\lambda_{c}^{'}l_{I}=\left[ \frac{f\left( \theta\right)\lambda_{c}^{'}}{\sqrt{3}}+\lambda_{c} \right]\frac{k\chi^{*}}{2}, \#(S7) \end{aligned}$$

with $f\left( \theta\right)=\left( 2+\cos\theta\right)/\sin\theta$. Submitting Eq (S7) into Eq (2) in the main text, we obtain the potential energy of a single cell as

$$\begin{aligned} {e(\lambda_{c},\lambda_{c}^{'},\theta)=\frac{1}{2}\left( \lambda_{c}\lambda_{c}^{'}k^{2}-1 \right)}^{2}+\frac{\kappa}{2}\left\{ \left[ \frac{f\left( \theta\right)\lambda_{c}^{'}}{\sqrt{3}}+\lambda_{c} \right]\frac{k\chi^{*}}{2}-\chi\right\}^{2}. \#(S8) \end{aligned}$$

Considering the stress-free state as the reference state, the nominal stresses of a single cell can be written as

$$s_{c}= \frac{e(\lambda_{c},\lambda_{c}^{'},\theta)}{k^{2}\partial\lambda_{c}}, s_{c}^{'}= \frac{e(\lambda_{c},\lambda_{c}^{'},\theta)}{k^{2}\partial\lambda_{c}^{'}}, (S9)$$

where $k^{2}$ is the cell area in the stress-free state. Besides, the energy minimization requires

$$\begin{aligned} \frac{\partial e(\lambda_{c},\lambda_{c}^{'},\theta)}{\partial\theta}=0.\#\left( S10 \right) \end{aligned}$$

Eq (S10) suggests $f^{'}\left( \theta\right)=0$, that is $\theta={120}^{^{\circ}}$. This means epithelial cells in this configuration also undergo isogonal deformation. Submitting $\theta={120}^{^{\circ}}$ into Eq (S9) leading to the following constitutive equation:

$$\begin{aligned} s_{c}=\lambda_{c}^{'}\left( k^{2}\lambda_{c}\lambda_{c}^{'}-1 \right)+\frac{\kappa\chi^{*}\left[ -2\chi+k\chi^{*}(\lambda_{c}+\lambda_{c}^{'}) \right]}{4k},\#(S11) \end{aligned}$$

$$\begin{aligned} s_{c}^{'}=\lambda_{c}\left( k^{2}\lambda_{c}\lambda_{c}^{'}-1 \right)+\frac{\kappa\chi^{*}\left[ -2\chi+k\chi^{*}(\lambda_{c}+\lambda_{c}^{'}) \right]}{4k}.\#(S12) \end{aligned}$$

For the uniaxial tensile test, the transverse stress $s_{c}^{'}=0$, and the nominal stress $s_{c}$can be determined as

$$\begin{aligned} s_{c}(\lambda_{c})=\frac{2\kappa\chi^{*}\left( \chi^{*}-2k\chi\lambda_{c}+k^{2}\chi^{*}{\lambda_{c}}^{2} \right)\left[ \kappa\chi^{*}\left( -\chi+k\chi^{*} \lambda_{c} \right)+2k \lambda_{c}\left( -1+k^{2}{\lambda_{c}}^{2} \right) \right]}{{k\left( \kappa{\chi^{*}}^{2}+4k^{2}{\lambda_{c}}^{2} \right)}^{2}},\#(S13) \end{aligned}$$

which has the same expression with the nominal stress defined in Eq (17). The uniaxial Cauchy stress is related to the nominal stress $s_{c}$ as $\sigma_{c}=s_{c}/\lambda_{c}^{'}$.

To derive the tissue stress-stretch relation from the cellular elastic response in Eq (S13), we still need to know the relationship between tissue stretch ratios ($\lambda$ and $\lambda^{'}$) and cellular stretch ratios ($\lambda_{c}$ and $\lambda_{c}^{'}$). As mentioned in the main text, we have ${\lambda_{c}=\lambda}_{T}^{+}\left( \lambda/\lambda_{T} \right)=n\lambda$ (with $n=4\sqrt{3}/9$). On the other hand, the transverse stretch ratios should satisfy $\lambda_{c}^{'}l_{I}=2\lambda^{'}w_{I}$, suggesting $\lambda_{c}^{'}=\sqrt{3}\lambda^{'}$. Then the tissue nominal stress $s(\lambda)$after topological transition yields

$$\begin{aligned} s\left( \lambda\right)=\sigma_{c}\lambda^{'}=\frac{\lambda^{'}}{\lambda_{c}^{'}}s_{c}\left( \lambda_{c} \right)=\frac{\sqrt{3}}{3}s_{c}\left( n\lambda\right) . \#\left( S14 \right) \end{aligned}$$

Submitting Eq (S13) into Eq (S14), we obtain the specific relationship between the tissue nominal stress $s$ and the tissue stretch ratio $\lambda$ after topological transition as

$$\begin{aligned} s =\frac{2\kappa\chi^{*}\left[ \chi^{*}-2k\chi n\lambda+k^{2}\chi^{*}({n\lambda)}^{2} \right]\left\{ \kappa\chi^{*}\left( -\chi+k\chi^{*}n\lambda\right)+2kn\lambda\left[ -1+k^{2}({n\lambda)}^{2} \right] \right\}}{\sqrt{3}k\left[ \kappa\chi^{*}+4k^{2}{(n\lambda)}^{2} \right]^{2}}.\#(S15) \end{aligned}$$
