# Supplementary material for: Necking of epithelial tissues with cellular topological transition
Source: PLoS Comput Biol. 2026 Jul 29;22(7):e1014565. doi: 10.1371/journal.pcbi.1014565 (PMC13436786; doi:10.1371/journal.pcbi.1014565)
Supplement: Supplementary file 6 [file pcbi.1014565.s001.docx]

**S1 Fig. Comparison of elastic stress-stretch curves between ordered and disordered tissues.**
